# X-ray imaging of a water bear offers a new look at tardigrade internal anatomy

**DOI:** 10.1186/s40851-019-0130-6

**Published:** 2019-05-11

**Authors:** Vladimir Gross, Mark Müller, Lorenz Hehn, Simone Ferstl, Sebastian Allner, Martin Dierolf, Klaus Achterhold, Georg Mayer, Franz Pfeiffer

**Affiliations:** 1 Department of Zoology, Institute of Biology, University of Kassel, Heinrich-Plett-Straße 40, 34132 Kassel, Germany; 2 Department of Physics and Munich School of BioEngineering, Technical University of Munich, 85748 Garching, Germany; 3 Department of Diagnostic and Interventional Radiology, Klinikum rechts der Isar, Technical University of Munich, 81675 Munich, Germany

**Keywords:** X-ray nanoCT imaging, Biological imaging, 3D reconstruction, Tardigrada

## Abstract

**Background:**

Tardigrades (water bears) are microscopic invertebrates of which the anatomy has been well studied using traditional techniques, but a comprehensive three-dimensional reconstruction has never been performed. In order to close this gap, we employed X-ray computed tomography (CT), a technique that is becoming increasingly popular in zoology for producing high-resolution, three-dimensional (3D) scans of whole specimens. While CT has long been used to scan larger samples, its use in some microscopic animals can be problematic, as they are often too small for conventional CT yet too large for high-resolution, optics-based soft X-ray microscopy. This size gap continues to be narrowed with advancements in technology, with high-resolution imaging now being possible using both large synchrotron devices and, more recently, laboratory-based instruments.

**Results:**

Here we use a recently developed prototype lab-based nano-computed tomography device to image a 152 μm-long tardigrade at high resolution (200–270 nm pixel size). The resulting dataset allowed us to visualize the anatomy of the tardigrade in 3D and analyze the spatial relationships of the internal structures. Segmentation of the major structures of the body enabled the direct measurement of their respective volumes. Furthermore, we segmented every storage cell individually and quantified their volume distribution. We compare our measurements to those from published studies in which other techniques were used.

**Conclusions:**

The data presented herein demonstrate the utility of CT imaging as a powerful supplementary tool for studies of tardigrade anatomy, especially for quantitative volume measurements. This nanoCT study represents the smallest complete animal ever imaged using CT, and offers new 3D insights into the spatial relationships of the internal organs of water bears.

**Electronic supplementary material:**

The online version of this article (10.1186/s40851-019-0130-6) contains supplementary material, which is available to authorized users.

## Background

The Tardigrada, commonly called water bears, consists of over 1300 species of eight-legged microinvertebrates that inhabit aquatic and semi-terrestrial habitats worldwide [1]. They are best known for their ability to enter a state of suspended animation known as cryptobiosis and thereby survive a wide range of extreme environmental conditions [2]. One of the characteristics that may enable this behavior is a reduction in surface area due to their extremely small body size [2], with the largest tardigrade species rarely exceeding 1 mm in length and most species reaching only 250–500 μm [1]. For microscopy and imaging, however, their small body size can make it difficult to investigate individual organs or structures. Although the overall anatomy of tardigrades has been studied in detail for many decades, one aspect that has remained elusive is a comprehensive, three-dimensional (3D) visualization of the entire tardigrade anatomy, including volume measurements of individual structures. Such a reconstruction is important in order to truly understand the spatial relationships between the structures inside the miniscule tardigrade body. In an effort to close this gap, we decided to apply X-ray computed tomography (CT): a technique that has emerged in recent years as the leading method for generating 3D scans of whole specimens.

X-ray computed tomography is non-invasive and is used to produce 3D datasets of samples based on the distribution of their attenuation coefficients, i.e., how easily the sample is penetrated by the X-ray beam. Continuing progress in the development of advanced X-ray sources, optics, and detectors in recent years has improved the resolution of this imaging technique to resolutions in the micrometer (=microCT) and, more recently, nanometer range (=nanoCT) [3]. At synchrotron radiation facilities, nanoCT beamlines based on advanced X-ray optics, such as Kirkpatrick-Baez optics or waveguides allow for CT imaging of relatively large samples with nanometer resolutions [4, 5]. In the lab, commercially available table-top devices using rotating anode generators at 8 keV combined with X-ray lenses can acquire CT data at resolutions down to 50 nm [6]. However, the specimens that can be imaged with such an instrument are often limited to a few tenths of a micrometer in size [6], which is smaller than most zoological samples. On the other hand, conventional laboratory-based microCT devices can image larger samples, but their limited resolutions (> 500 nm) hamper the detailed visualization of structures at the nanometer level, which is necessary for the investigation of very small animals, such as rotifers, tardigrades, and many nematode species. In fact, most of the animal diversity on our planet, including meiofauna and zooplankton, falls into this range [7, 8]. Despite these traditional limitations, a recent study on the nematode *Caenorhabditis elegans* was able to image a sample of ~ 1 mm in length [9]. In addition, two proof-of-concept experiments scanned tardigrades in order to demonstrate a new phase-retrieval method [10] or the advantages and disadvantages of a high-throughput μCT setup [11], both performed at synchrotron facilities. However, an in-depth analysis of the internal anatomy was not performed in either case.

Recently, a prototype nanoCT setup was introduced in one of our laboratories [3] that can achieve resolutions down to 100 nm while still offering the possibility of measuring relatively large samples, thereby opening up the potential for detailed imaging of micrometazoans that are too large for optics-based X-ray microscopy but too small for conventional CT techniques. We used this new nanoCT setup to analyze the tardigrade *Hypsibius exemplaris*, one of the smallest metazoans (multicellular animals). We chose *H. exemplaris* because this species has been used as a model for studying many aspects of tardigrade biology and evolution (reviewed in [12, 13]) and is the first tardigrade species to have its complete genome sequenced [14, 15]. In this study, we were able to segment the majority of internal structures, and the resulting dataset allowed us to measure the segmented volumes directly without having to rely on geometrical approximations. Our results represent the smallest whole animal imaged to date using CT and offer a new quantitative, 3D perspective of tardigrade anatomy.

## Results

**Fig. 1:**
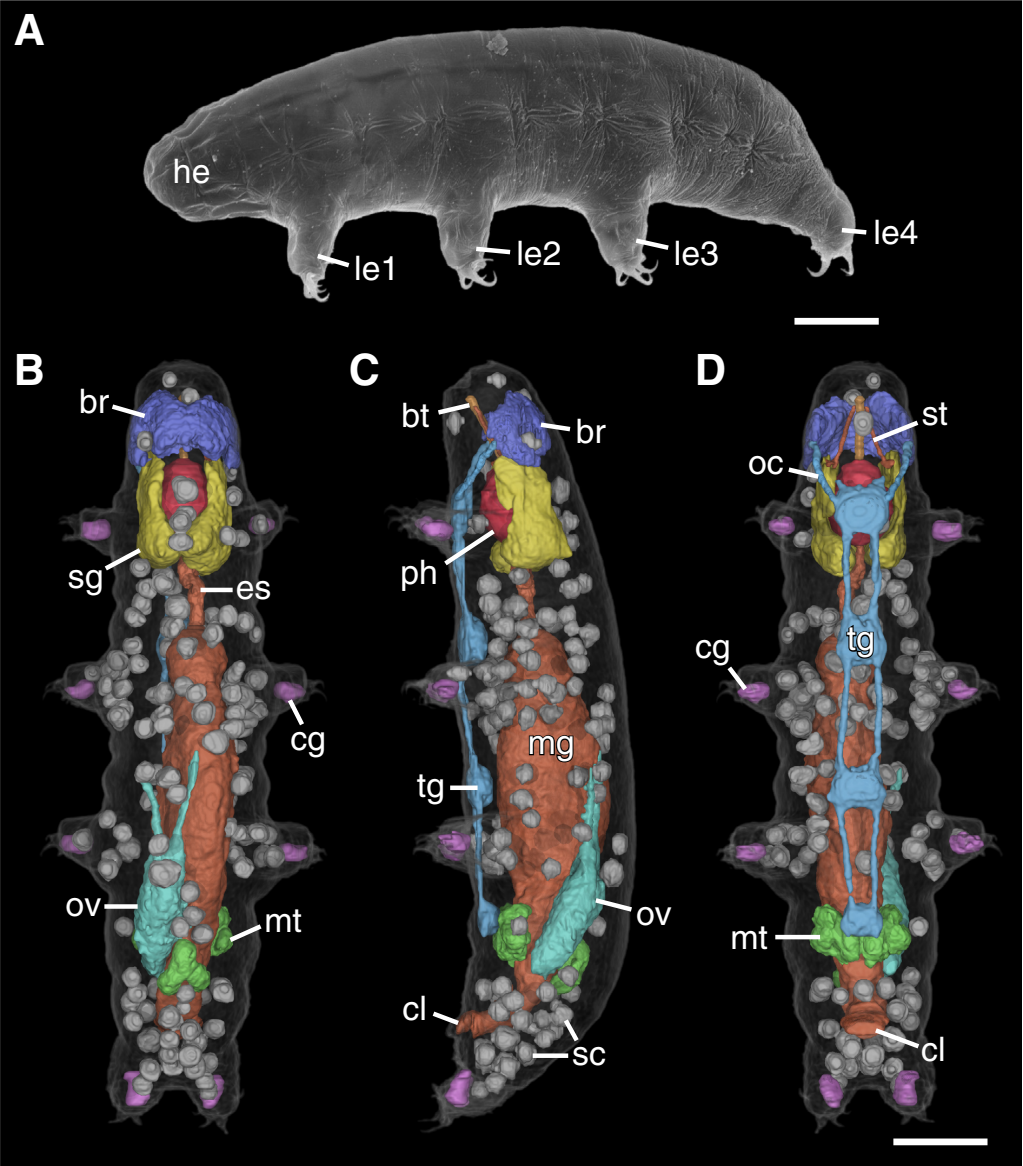
The tardigrade *Hypsibius exemplaris*. **a** Scanning electron micrograph of an adult specimen. Lateral view; anterior is left, dorsal is up. **b**-**d** 3D renderings of X-ray nanoCT data showing the entire body and all segmented organs in dorsal (**b**), lateral (**c**), and ventral (**d**) views. Anterior is up. Note the large salivary glands (yellow), the ovary and its two anterior ligaments (teal), and the distribution of storage cells (gray) throughout the body. Abbreviations: br, brain; bt, buccal tube; cg, claw glands; cl, cloaca; es, esophagus; he, head; le1–le4, legs one to four; mg, midgut; mt, Malpighian tubules; oc, outer connectives; ov, ovary; ph, pharynx; sg, salivary glands; sc, storage cells; tg, trunk ganglion. Scale bars: 20 μm (scale bar in **d** for **b**-**d**)

**Fig. 2:**
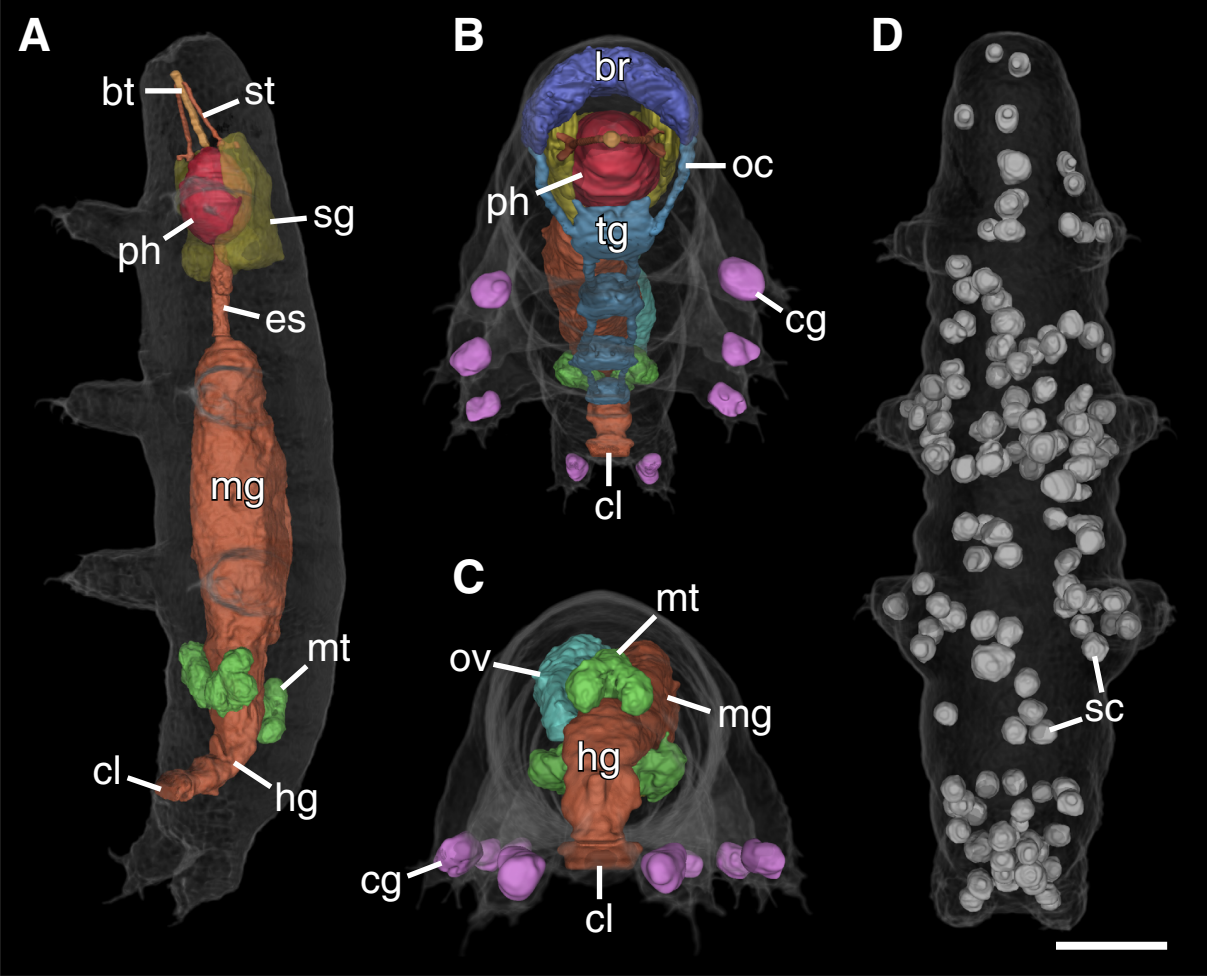
3D renderings of selected structures of the tardigrade *Hypsibius exemplaris*. **a** The digestive system and associated structures. Ventrolateral view; anterior is up. The large midgut is the most prominent region of the digestive tract. Note how the large salivary glands (yellow) surround the pharynx (red). The transition between the midgut and hindgut is marked by the attachment sites of the Malpighian tubules (green). **b** Frontal-ventral view emphasizing the organization of the buccopharyngeal apparatus. **c** Posterior view showing the orientation of the ovary (teal) and Malpighian tubules (green). Notice how the ovary is located to the left of the midgut, displacing the midgut to the right side of the midline of the body. The Malpighian tubules consist of paired ventral units and an unpaired dorsal unit. **d** The 137 storage cells of this specimen are distributed throughout the body. Dorsal view; anterior is up. Abbreviations: bt, buccal tube; br, brain; cg, claw glands; cl, cloaca; es, esophagus; hg, hindgut; mg, midgut; mt, Malpighian tubules; oc, outer connectives; ov, ovary; ph, pharynx; sc, storage cells; sg, salivary glands; st, stylet. Scale bar: 20 μm (for **a**-**d**)

**Fig. 3:**
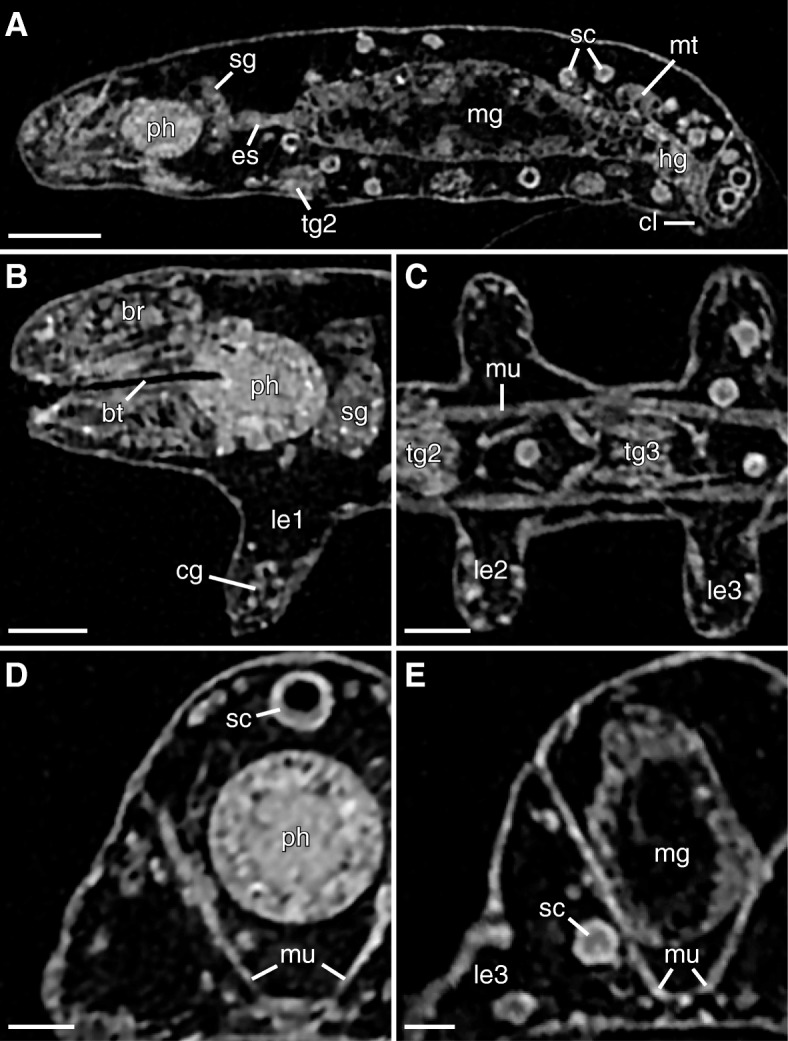
NanoCT slices of the tardigrade *Hypsibius exemplaris*. Anterior is left (in **a**-**c**), dorsal is up (in all images except for **c**). The slices have a voxel size of 270 nm (in **a**, **c** and **e**), and 200 nm (in **b** and **d**), respectively. **a** Sagittal view through the midline of the body showing the digestive tract. The transition between the midgut and hindgut is marked by the attachment sites of the Malpighian tubules. **b** Sagittal view through the head region showing the pharynx and the lumen of the buccal tube. **c** Horizontal view through the ventral body (at the level of the ganglia) showing the second and third leg pairs. The trunk ganglion in each segment lies anterior to the legs of the same segment. Notice the thick ventral longitudinal muscles. **d** Transverse view through the head region showing the pharynx, a large storage cell, and paired dorsoventral muscles. **e** Transverse view through the third trunk segment showing the large midgut and paired dorsoventral muscles. Notice how the storage cell labeled in **e** shows a relatively homogeneous gray value while that in **d** appears hollow. Abbreviations: br, brain; bt, buccal tube; cg, claw gland; cl, cloaca; es, esophagus; hg, hindgut; le1–le3, legs one to three; mg, midgut; mt, Malpighian tubules; mu, muscle; ph, pharynx; sc, storage cells; sg, salivary glands; tg1–tg2, trunk ganglia one and two. Scale bars: 20 μm (in **a**), 10 μm (in **b** and **c**), 5 μm (in **d** and **e**)

The *Hypsibius exemplaris* specimen presented in this study was imaged in its entirety with an isotropic voxel size of 270 nm. It was reconstructed with a statistical iterative reconstruction algorithm [3, 16]. Since the head region of the tardigrade contains minute structures that could not be resolved in sufficient detail at 270 nm voxel size, the head was rescanned with a voxel size of 200 nm and the obtained data were used to segment the structures of the head region. Subsequently, the obtained label fields were merged with the segmentation data from the whole-body measurements and the combined dataset was used for the presented visualizations and volume calculations. The specimen is 152 μm in length from anterior to posterior (not including the fourth pair of legs), 32 μm in maximum width (measured between the third and fourth pairs of legs), and 0.14 nl in volume including the skin and cuticle (Figs. 1b-d, 2a-d and 3a, c). The volumes of individual structures are presented as a percentage of the total body volume (TBV; Table 1).

**Table 1:** Volumes of the segmented structures given in cubic micrometers and as a percentage of the total body volume (TBV)

Structure	Volume (μm^3^)	Volume (% TBV)
Total body volume	137,414.81	100.00
Midgut + hindgut	13,705.79	9.97
Storage cells^a,b^	6599.35	4.80
Salivary glands^a^	2273.69	1.65
Pharynx	1770.55	1.29
Brain	1392.90	1.01
Ovary	1325.64	0.96
Malpighian tubules^a^	1133.05	0.82
Claw glands^a,c^	711.34	0.52
Esophagus	162.06	0.12

^a^Given volume represents the total of all individual structures

^b^For volume distribution of individual cells, see Fig. 4

^c^Individual claw glands range in volume from 66.8–107.6 μm^3^

The CT scans revealed the overall structure and spatial relationships of all major organs in the body (Figs. 1b-d and 2a-d; Additional file 2). The majority of structures were manually segmented in their entirety, including the salivary glands (Figs. 1b-d, 2a, b and 3a, b, d; Additional file 1a, b), ovary (Figs. 1b-d and 2c), Malpighian tubules (Figs. 1b-d, 2a, c and 3a), and the entire digestive system with buccopharyngeal apparatus (Figs. 1b-d, 2a-c and 3a, b, d, e). The major parts of the nervous system (i.e., the brain, trunk ganglia, and their connectives) were also segmented, although the inner connectives between the brain and first trunk ganglion as well as peripheral and leg nerves were not identified (Figs. 1b-c, 2b, c and 3a-c; Additional file 1a, b). The dorsal, saddle-shaped brain (1.0% TBV) is revealed to be in close proximity to the salivary glands (1.7% TBV), which occupy most of the space surrounding the pharynx (1.3% TBV), especially dorsally (Figs. 1b-d and 2b; Additional file 1a, b). The ganglion of each segment is positioned anterior to the legs of the same segment (Figs. 1c, d and 3c). A lateral view reveals that the connectives traverse through the ventral side of each ganglion (Fig. 1c). The outer connectives linking the brain to the first trunk ganglion run along the lateral wall of the head (Figs. 1c, d and 2b). All major neural structures described here, whether ventral or dorsal, lie adjacent to the epidermis (Figs. 1b-d, 2b and 3a).

The digestive system (Fig. 2a) is dominated by the midgut, which shows a highly folded lumen (Fig. 3a). A narrow esophagus (0.1% TBV) connecting the midgut to the pharynx winds between the salivary glands (Figs. 1b, 2a and 3a). The transition from midgut to hindgut could only be identified by the position where the Malpighian tubules attach; the midgut and hindgut were therefore segmented together and occupy 10.0% TBV (Figs. 1a-d, 2a, c and 3a). The digestive tract ends in a cloaca, located medially between the third and fourth leg pairs (Figs. 1c, d, 2a-c and 3a). The Malpighian tubules consist of a paired ventrolateral unit and an unpaired dorsal unit (together 0.8% TBV; Figs. 1b-d and 2c). The ventrolateral units lie in close proximity to each other on the ventral side (Fig. 1c). The ovary (1.0% TBV) is located dorsal and to the left side of the midgut, displacing the midgut to the right of the midline of the body (Fig. 2c, Additional file 2). There are two anterior ligaments connecting the ovary to the body wall (Fig. 1b, c). The left ligament attaches to the dorsolateral body wall while the right ligament runs along the dorsal midgut and attaches to the dorsal body wall (Fig. 1b, c).

**Fig. 4:**
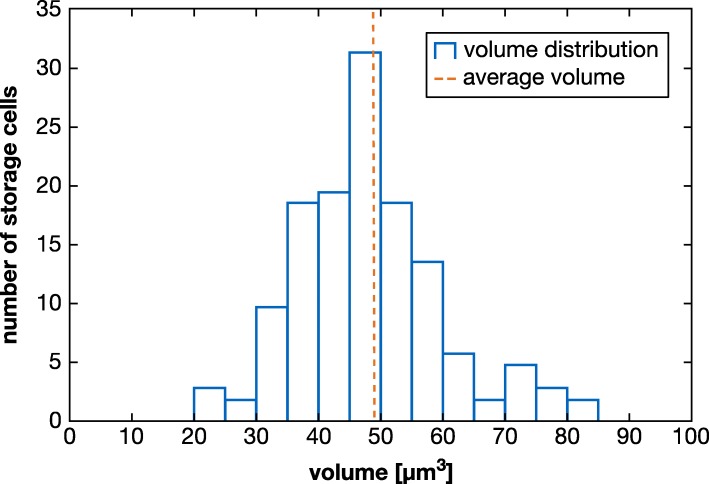
Distribution of storage cell volumes. The blue bars depict the volume distribution of the 137 storage cells in the investigated specimen for a bin size of 5 μm. The dashed orange line indicates the average volume of 48.2 μm^3^

Additionally, all storage cells in the body could be segmented individually (Figs. 1b-d and 2d). In the specimen presented in this study, there are a total of 137 storage cells (Figs. 1b-d and 2d). The storage cells measure 20.8–83.4 μm^3^ in volume (average of 48.2 μm^3^), together occupying 4.8% of the total body volume (Table 1; Fig. 4). Some storage cells appear hollow while others are relatively homogeneous in gray value (Fig. 3a, c–e). Although the storage cells are distributed throughout the body, they appear to be at a higher concentration in large areas of open body cavity, such as between the midgut and salivary glands and posterodorsal to the hindgut (Fig. 1b-d and 2d). There are two storage cells in close proximity to the brain (Fig. 1b, c). On the other hand, there are relatively few storage cells in the legs, where much of the distal region is occupied by the claw glands (88.9 μm^3^ each, together 0.5% TBV; Figs. 1b-d, 2b, c and 3b). The body musculature was also revealed by the CT scans (Fig. 3c-e), but the individual muscle cells were not segmented in this study.

## Discussion

### Technical remarks

For many arthropods, which possess sclerotized cuticular structures, fixation without additional contrasting is sufficient for quality CT imaging (see e.g. [17, 18]). In the present study, however, we tailored the protocol to maximize the overall contrast of a soft biological specimen that is normally not amenable to tomographic imaging. To achieve this, we used a sample preparation protocol based on a combination of techniques established for transmission and scanning electron microscopy. For post-fixation and contrasting, it has been shown that for onychophorans (a related group of soft-bodied invertebrates), OsO_4_ provides the highest level of overall tissue contrast compared to other contrasting agents [19]. In this regard, it is advantageous compared to selective stains, like those required for fluorescence microscopy, when visualizing the overall internal anatomy of a whole animal. We further enhanced the OsO_4_-contrasting steps of the protocol by adding ferrocyanide [20] and using the OTO technique (osmium-thiocarbohydrazide-osmium) [21].

Due to the advanced staining procedure applied in this work, the filtered backprojection (FBP) reconstruction of the tardigrade specimen (Additional file 1a) already showed a strong soft-tissue contrast. Furthermore, the introduced phase contrast, i.e., the edge enhancement caused by Fresnel diffraction, was small enough in the obtained data that it did not hamper the subsequent image segmentation steps. Consequently, it was not necessary to apply a phase-retrieval algorithm to improve the soft-tissue contrast, as had been demonstrated for the onychophoran limb [3]. This offers the advantage of no undesired image blurring, an artefact common to such phase-retrieval steps that typically must be removed by additional processing steps [3].

Instead, we used an edge-preserving statistical iterative reconstruction (SIR) that additionally sharpens the images by compensating for the source blurring during acquisition by either directly including a model for the source in the SIR [22] or by a separate deconvolution step [23]. The SIR reconstruction (Additional file 1b) clearly improved the image noise and resolution compared to the FBP reconstruction, thereby facilitating a precise segmentation and the visualization of fine details, such as the salivary glands and especially the brain, which could be more easily differentiated from the surrounding tissue (arrows in Additional file 1a, b).

On the one hand, the lack of a selective stain complicates the manual segmentation of structures that are closely associated with each other or are located in an area of high tissue density, such as the brain and associated structures in the tardigrade head as well as fine musculature. For this reason, the inner connectives could not be reliably segmented in our dataset, nor could the eyes be identified because they lie inside the contours of the brain [24]. On the other hand, lumens of structures such as the midgut and buccal tube have been well preserved during fixation and are easily recognizable in the CT scans. Further studies may focus on selectively enhancing the contrast of specific organs or structures by treating the sample with various contrasting agents [19].

### Morphological analysis

The quantitative nature of the data allowed us to measure the size and volume of most of the major structures in the body and analyze their spatial relationships in an animal that was not cut, punctured, or otherwise physically disrupted. The tardigrade *Hypsibius exemplaris* shown herein represents a relatively small specimen at 152 μm, while adult individuals typically measure ~ 230 μm in length [25]. The presence of an ovary suggests that this specimen was at least approaching sexual maturity, although any evidence of developing eggs is absent. Therefore, we estimate that the specimen presented herein is probably a young adult, since tardigrades generally continue growing even after sexual maturity [1].

*Hypsibius exemplaris* is a parthenogenetic species, meaning all individuals are females and possess an ovary. The ovary occupies 1.0% TBV in the investigated specimen, but we expect the size of this organ to vary greatly depending on the reproductive cycle of the animal [26, 27]. Interestingly, our scans show that the ovary develops not directly dorsal to the midgut, but rather dorsolaterally, thereby displacing the midgut. The attachment sites of the two anterior ligaments that connect the ovary to the body wall likewise reflect this asymmetry. While the ligaments are generally presented in the literature as isolated within the body cavity [28], the right ligament in our scans appears closely associated with the midgut wall. Whether the ovary always occupies the space to the left of the midgut or whether this position varies between tardigrades has, to our knowledge, never been addressed, as the position of the ovary is usually described simply as “dorsal” or overlying the midgut [28]. Future studies using nanoCT may also aim to quantify exactly how the volume of the ovary changes throughout the reproductive cycle. The same applies to the midgut, the size of which may depend not only on the nutritional state of the animal but also on the stage of the life cycle [26]. For example, tardigrades undergoing a molt are unable to feed due to the expulsion of the entire foregut (the so-called “simplex” stage), and the midgut shrinks in size accordingly [1, 27].

The nervous system in tardigrades consists of a dorsal brain and a ganglionated ventral nerve cord (reviewed in ref. [29]). Our volume measurements of the brain revealed that the size of this organ relative to body volume is approximately double that of honeybees and even greater compared to larger insects, such as diving beetles (ditystids; [30]). Our results therefore seem to follow the trend described by Haller’s Rule, by which a reduction in body size accompanies an increase in the relative brain volume, at least in insects [31]. However, direct comparisons between the brains of tardigrades and insects should be made with caution, as several species of featherwing beetles (ptilids), which are 380–630 μm in length, have brain volumes of 2.9–4.3% TBV [32], i.e., much larger than that of the tardigrade presented herein. Brain size may also depend in part on the innervation of any sensory organs on the head, as the specialized brain regions associated with these structures may be enlarged [33]. In this regard, it would be interesting to compare the brain sizes across different heterotardigrades, which possess an array of head appendages that are not present in eutardigrades like *H. exemplaris*.

Although we did not measure the volumes of the ventral trunk ganglia, analyzing these structures in 3D demonstrates that most of the cell bodies of each ganglion lie dorsal to the neuropil (i.e., connectives) [34]. This is different from onychophorans and pycnogonids, where most of the somata occupy the ventral space of the nerve cord and ganglion, respectively [35, 36]. The ganglia in arthropods have a more complex organization with neuropils at several levels [37] but share with tardigrades an anterior position relative to the corresponding leg pair of the same segment [38, 39]. This anterior shift, known as “parasegmental” (summarized in ref. [29]), is clearly visible in our scans.

The storage cells of tardigrades are cells of the body cavity that are involved primarily in nutritional maintenance [40, 41] but also play a role in vitellogenesis [42, 43] and possibly immune function [44]. These cells may therefore display variable internal morphologies and ultrastructure relating to their roles [41, 45], a hypothesis that our data support based on the different internal gray values and textures seen in the storage cells in our CT scans. The sizes of storage cells have been measured in several studies in a number of different species [40, 42, 45, 46]. Reuner et al. [40] performed the most comprehensive comparison (although *H. exemplaris* was not one of the species studied) and found that the number and size of storage cells varies greatly depending on the species. Assuming a spherical shape, the average diameter of the storage cells in our specimen is ~ 4.5 μm, thereby most closely resembling those of *Macrobiotus sapiens* in size, which measured 4.4–8.3 μm in diameter [40].

Previous studies have also investigated how different physiological processes affect storage cells, for example oogenesis [41–43, 47], cryptobiosis [46], and starvation [40]. Based on these studies, it is evident that many parameters affect different species differently. For example, anhydrobiosis (desiccation-induced cryptobiosis) has a significant effect on the average size of storage cells in *Milnesium tardigradum*, and *Richtersius coronifer* but not in *Paramacrobiotus tonollii* or *Macrobiotus sapiens* [40, 46]. On the other hand, starvation led to a decrease in the size of storage cells in all species investigated, supporting the hypothesis that the primary role of these cells is nutritional maintenance [40, 41].

The specimen presented herein has a smaller number and smaller size of storage cells than any species previously investigated using other techniques, as well as a relatively small body size [40]. Although the data of Reuner et al. [40] argue against a positive correlation between body size and the number of storage cells, our results seem to suggest otherwise (at least for *H. exemplaris*). It should also be noted that the diameter estimates of storage cells presented herein are based on volume measurements; we did not directly measure diameters as has generally been done in previous studies. Because a large proportion of storage cells tend to be irregular in shape [46], calculations based solely on diameter may be imprecise compared to volume measurements. The different techniques may therefore lead to significant differences in size estimates. For example, while critical point drying as performed in the present study may affect the absolute sizes of all structures, our data indicate that, even if structures did indeed shrink, the degree of shrinkage was similar for all structures and spatial relationships were thus unaffected [48]. Hence, our measurements based on % TBV are intended to account for this potential artifact. Although future studies may aim to specifically address the effects of different methods on final size estimates, we propose that CT scans may be a more accurate method of analyzing size distributions of storage cells. An additional advantage is that the scanned specimens remain fully intact, minimizing the chance that any storage cells may have leaked out of the animal.

## Conclusion

Previous work based on, for example, confocal laser-scanning microscopy [41, 49] or a series of sections [50] provided three-dimensional representations only of select structures of tardigrades. In contrast, CT offers an isometric, 3D visualization of a tardigrade and its internal organs without introducing sectioning artifacts. The dataset resulting from our experiments allowed for the direct measurement of segmented volumes without having to rely on geometrical approximations, thereby enabling a higher accuracy of volume measurements. This opens the door to possible future investigations of the changes that occur during the formation of tuns (resistant states), for example, which is accompanied by a large reduction in the volume and surface area of the body [2]. These results therefore continue the trend of tomographic imaging playing an increasingly larger role in morphological studies and demonstrate its potential for investigating microinvertebrates.

## Methods

### Specimen preparation

Specimens of the eutardigrade *Hypsibius exemplaris* Gąsiorek et al., 2018 (previously “*H. dujardini* Doyère, 1840”) were kept in plastic Petri dishes filled with mineral water (Volvic, Danone Waters Deutschland, GmbH, Frankfurt am Main, Germany) at 21 °C and fed unicellular algae (*Chlorococcum* sp.). Live specimens were asphyxiated at 60 °C for 30 min and fixed in 2.5% glutaraldehyde buffered with PBS (0.1 mol l^− 1^ phosphate-buffered saline, pH 7.4) at 4 °C for at least 1 h. Samples were washed 5 × 10 min with cold PBS and post-fixed in an aqueous solution containing 1% OsO_4_ + 1% potassium ferrocyanide (Merck KGaA, Darmstadt, Germany) at 4 °C for 1 h. After 5 × 10 min washes with distilled water, samples were incubated in 1% thiocarbohydrazide (Sigma-Aldrich, St. Louis, MO, USA) for 20 min at room temperature (RT). After another 5 × 10 min washes with distilled water, samples were incubated overnight in 1% OsO_4_ at RT. On the following day, samples were washed 5 × 10 min with distilled water at RT and dehydrated through an ethanol series (5 min each at 20, 50, 70, 90, and 2 × 100% ethanol). The specimens were then transferred in absolute ethanol to a capsule with a pore size of 78 μm (Plano GmbH, Wetzlar, Germany) and critical point dried using a Bal-Tec 030 Critical Point Dryer (Balzers, Liechtenstein). Individual tardigrades were mounted manually on hedgehog quills using superglue.

For scanning electron microscopy, adult tardigrades were asphyxiated as described above and fixed in 4% PBS-buffered formalin for at least 1 h at RT. After 2 × 15 min washes with PBS, the specimens were dehydrated in an ethanol series and critical point dried as described above. Samples were attached to aluminum stubs with double-sided carbon tape, sputter-coated with gold-palladium using a Polaron SC7640 sputter coater (Quorum Technologies, Kent, UK), and imaged using a Hitachi S-4000 field emission scanning electron microscope (Hitachi High-Technologies Europe GmbH, Krefeld, Germany) at an accelerating voltage of 10 kV and a working distance of 15 mm.

### NanoCT imaging

The technological details of the nanoCT system have previously been described in detail [3], and are only briefly reviewed here. The main components of this system are a nanofocus X-ray source (NanoTube, Excillum, Sweden), a single-photon counting detector (Pilatus 300 K-W, Dectris, Switzerland) [51] and a high-precision air-bearing rotary stage (RT150S, LAB Motion Systems, Belgium). The system does not include any X-ray optics and is based on the principle of geometrical magnification, i.e., the sample is illuminated by an X-ray point source and the projection image of the sample is magnified onto the X-ray detector, whereby the magnification factor is only depending on the ratio between the source-to-detector distance and the source-to-rotation-axis distance. By acquiring a series of magnified projection images at different rotation angles, CT data with resolutions down to 100 nm can be generated [3]. Sixteen datasets (nine of the whole body and seven from the head region) were generated from five individual animals, for a total of 39 separate measurements. The datasets were evaluated qualitatively and that of the highest quality was chosen for subsequent segmentation, presented herein (Additional files 3, 4, 5 and 6). All presented CT data were acquired at a peak voltage of 60 kV with 1599 projections distributed over 360°. The X-ray camera was operated at a threshold of 2.7 keV and the exposure time per image was 2 s, resulting in a total acquisition time per CT measurement of 2 h and 15 min.

Since the animal was too large to be fully imaged in a single CT scan at the desired voxel size of ~ 300 nm, we performed four separate CT measurements at different vertical positions with a voxel size of 270 nm and combined the data to a single volume (Additional file 3). The source-to-rotation-axis distance and the source-to-detector distance for these measurements were 0.71 mm and 450 mm, respectively. The additional scan of the head region was performed at a source-to-rotation-axis distance of 0.61 mm and a source-to-detector distance of 520 mm, resulting in an effective voxel size of 200 nm (Additional file 5). The X-ray spot size of the nanofocus source was between 400 and 500 nm for all presented measurements. Considering previous results from this nanoCT system [3] and the fact that we correct for blurring of the source in the applied processing, the effective resolution in the final data was estimated to be very close to the effective voxel size.

### Data and image processing

To achieve an isotropic background intensity, the acquired projection images were normalized with a corresponding flat-field image, i.e., an image of the illumination of the detector without the sample in the beam. For the dataset of the entire tardigrade, the projections were pre-processed with a Richardson-Lucy deconvolution algorithm [23] and reconstructed with an edge-preserving SIR as described previously [3].

For the tomographic reconstruction of the head region, a statistical iterative reconstruction algorithm was used, which explicitly models the blurring of the source, the attenuation of the X-rays as well as their statistical properties [22, 52]. Thereby, the blurring of the source, which is the predominant contribution of the intrinsic image blur of the system, was estimated to be a Gaussian blur with a full width half maximum of 500 nm. For regularization, an edge-preserving Huber penalty [53] was chosen, which takes the nearest 26 neighbors into account. The transition parameter of the Huber penalty was estimated from the background noise level of the corresponding filtered backprojection reconstruction. The regularization strength was chosen empirically to give a good tradeoff between noise and resolution. The L-BFGS algorithm was used as the optimization routine [54].

Segmentation of the two datasets (whole body and head region) was done manually per slice using the brush tool and an intensity mask in the open source software package Fiji ImageJ [55] and Amira 5.4.0 (Thermo Fischer Scientific, Hillsboro, Oregon, USA). The label fields of the head region of the whole-body dataset were replaced by the labels of the higher resolution dataset by applying a registration algorithm with an affine transformation model. The resulting combined dataset was then used for all subsequent analyses. The volumetric analysis of the segmented organs was performed with Avizo Fire 8.1 (Thermo Fischer Scientific, Hillsboro, Oregon, USA). Volume renderings were generated with VGStudioMAX 3.1 (Volume Graphics GmbH, Heidelberg, Germany) and panels were assembled and labeled using Illustrator CS6 (Adobe Systems Incorporated, San Jose, CA, USA). Additional file 2 was edited using Adobe Premier Pro CS6.

## Additional files

Additional file 1: Processing of nanoCT data from the tardigrade *Hypsibius exemplaris*. Sagittal view through the head region showing the improvement in sharpness of, e.g., the salivary glands (blue arrows) and especially the brain (orange arrow). (**a**) Standard filtered backprojection reconstruction. **(b)** Statistical iterative reconstruction with an integrated model of the source. The reconstructed voxel size is 200 nm in (**a**) and (**b**). Scale bars: 10 μm (in both images). (PDF 1080 kb)

Additional file 2: Animated 3D rendering of the tardigrade *Hypsibius exemplaris* showing all segmented structures and a fly-through of the original nanoCT volume. (MP4 30928 kb)

Additional file 3: Image stack showing the processed scan of the whole body of *Hypsibius exemplaris* used for segmentation in this study. (3D-TIF 48795 kb)

Additional file 4: Label fields showing the results of the segmentation of the whole-body dataset presented in this study. (3D-TIF 1627 kb)

Additional file 5: Image stack showing the processed scan of the head region of *Hypsibius exemplaris* used for segmentation in this study. (3D-TIF 252942 kb)

Additional file 6: Label fields showing the results of the segmentation of the head region dataset presented in this study. (3D-TIF 2283 kb)
