# Supplementary figures and images for: X-ray imaging of a water bear offers a new look at tardigrade internal anatomy
Source: Zoological Lett. 2019 May 11;5:14. doi: 10.1186/s40851-019-0130-6 (PMC6511223; doi:10.1186/s40851-019-0130-6)

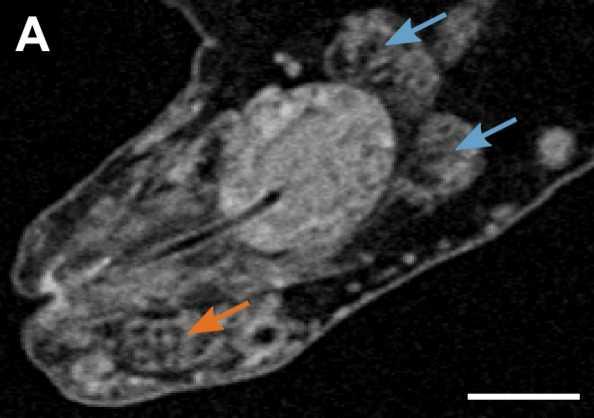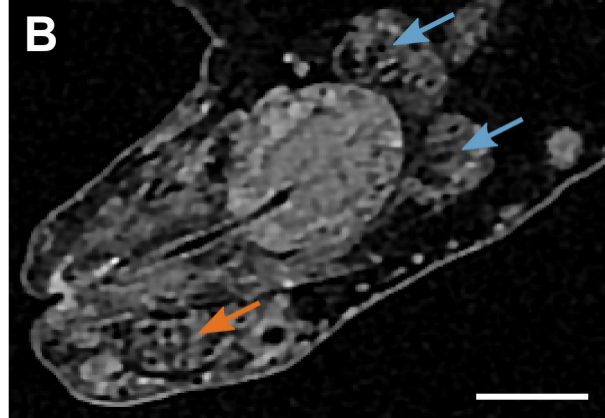

Supplement: Supplementary file 1 — Processing of nanoCT data from the tardigrade Hypsibius exemplaris. Sagittal view through the head region showing the improvement in sharpness of, e.g., the salivary glands (blue arrows) and especially the brain (orange arrow). (a) Standard filtered backprojection reconstruction. (b) Statistical iterative reconstruction with an integrated model of the source. The reconstructed voxel size is 200 nm in (a) and (b). Scale bars: 10 μm (in both images). (PDF 1080 kb) [file 40851_2019_130_MOESM1_ESM.pdf]

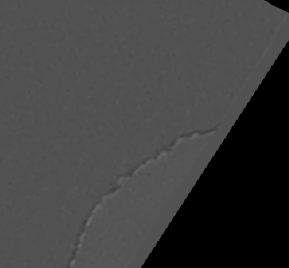

Supplement: Supplementary file 3 — Image stack showing the processed scan of the whole body of Hypsibius exemplaris used for segmentation in this study. (3D-TIF 48795 kb) [file 40851_2019_130_MOESM3_ESM.tif]
